# Genome-Wide Expression Quantitative Trait Loci Analysis Using Mixed Models

**DOI:** 10.3389/fgene.2018.00341

**Published:** 2018-08-21

**Authors:** Chaeyoung Lee

**Affiliations:** Department of Bioinformatics and Life Science, Soongsil University, Seoul, South Korea

**Keywords:** expression quantitative trait locus, genetic association, genetic variance, heritability, mixed model

## Abstract

Expression quantitative trait loci (eQTLs) are important for understanding the genetic basis of cellular activities and complex phenotypes. Genome-wide eQTL analyses can be effectively conducted by employing a mixed model. The mixed model includes random polygenic effects with variability, which can be estimated by the covariance structure of pairwise genomic similarity among individuals based on genotype information for nucleotide sequence variants. This increases the accuracy of identifying eQTLs by avoiding population stratification. Its extensive use will accelerate our understanding of the genetics of gene expression and complex phenotypes. An overview of genome-wide eQTL analyses using mixed model methodology is provided, including discussions of both theoretical and practical issues. The advantages of employing mixed models are also discussed in this review.

## Introduction

Gene expression is the frontier process linking genotypes to phenotypes, and thus the genetics of gene expression is critical for dissecting the genetic basis of complex phenotypes. Currently, the genetics of gene expression largely depends on identifying an expression quantitative trait locus (eQTL), i.e., an association between gene expression and the genotype at a locus. Genome-wide eQTL studies have shown that the eQTLs explain a substantial proportion of variation in gene expression (Spielman et al., 2007); about 90% of the variation in the expression of many genes has been attributed to nucleotide variants (Yang S. et al., 2014). Genome-wide eQTL analyses have enabled us to obtain a profile of regulatory signals for each gene and to compare multiple profiles for cells with different functions. Furthermore, eQTL analyses for a variety of molecular traits can provide evidence for the specific regulatory stages and functions of gene expression. Data production for such eQTL analyses is increasing dramatically with the continuous development of technology. The Geuvadis consortium generated RNA sequencing data on lymphoblastoid cell lines of 462 individuals from the 1000 Genome Project (Lappalainen et al., 2013), and the Genotype-Tissue Expression consortium reported RNA sequencing data on 1641 samples across 43 tissues from 175 individuals (GTEx Consortium, 2015). The choice of statistical method for analyzing these data is increasingly important to draw better inferences.

Mixed model methodology is an emerging method for genome-wide association studies (GWASs); it was originally applied to the genome-wide identification of loci associated with a phenotypic trait but can be extended to analyses of associations between loci and intermediate molecular traits, such as RNA and protein expression levels. The mixed model methodology has been employed for nearly a half century for genetic analyses because it can explain polygenic effects while this is the intractable problem using fixed models. The polygenic effects can be assessed as random effects which are the special feature of mixed models, using pedigree-based genetic relationships. Currently, GWAS data are evaluated by mixed models with genomic similarity among unrelated individuals modified from pedigree information to nucleotide variant information. The direct approach of the genetic difference among individuals is an efficient way of avoiding population stratification that is one of the critical problems producing spurious genetic associations in GWAS. An overview of eQTL analyses by the mixed model methodology is provided, with an emphasis on important issues. Only essential mathematical notation for analytical models and relevant estimation methods are concisely presented in this review to ensure a clear presentation of mixed models and to avoid the intricacies of specific conditions.

## Historical Look at Mixed Models

Henderson (1950, 1953) developed the mixed model and the corresponding parameter estimation method for applications in genetics. Prior to its development, Fisher (1925) estimated variance components using mean squares of analysis of variance (ANOVA) and their expected values, but this estimation is limited to balanced data. Henderson’s mixed model methodology has been utilized extensively for the genetic improvement of animals. Since the mixed model is hierarchical, the estimation of variance components for fixed and random effects is stressed as a priority. Various estimation methods for variance components have been applied, and they can be categorized into four general types: ANOVA-based estimation, distribution-free quadratic estimation, likelihood-based estimation, and Bayesian estimation (**Table 1**). An example of distribution-free quadratic estimation is the minimum variance quadratic unbiased estimation (MIVQUE), in which a local best unbiased estimate is obtained with minimum variance of the quadratic form of a random variable (Rao, 1971). Empirical bias was observed in the application of the MIVQUE to genetic and residual variance components (Van Tassell et al., 1995). Likelihood-based estimation has attractive statistical properties, such as asymptotic unbiasedness and asymptotic efficiency (Casella and Berger, 1990). Nevertheless, restricted maximum likelihood (REML; Patterson and Thompson, 1971) estimation has been dominantly preferred to maximum likelihood (ML; Hartley and Rao, 1967) estimation. This is because the degrees of freedom in estimating fixed effects are explained for REML, but not for ML. Empirical unbiasedness of REML estimates has been verified using simulated data, even for artificial selection in animals (Jensen and Mao, 1991; Lee and Pollak, 1997b). REML has been utilized as the standard method for estimating variance components in mixed model analyses. Representative algorithms for obtaining REML estimates include the quasi-Newton method (Kennedy and Gentle, 1980), average information method (Johnson and Thompson, 1995), expectation maximization method (Laird et al., 1987), and derivative-free method (Boldman and Van Vleck, 1991). The Bayesian estimation of variance components is feasible by Markov chain Monte Carlo (MCMC), a numerical procedure for sampling from a desired probability distribution at equilibrium in a Markov chain. Bayesian estimation is increasingly used for variance component estimation. The advantages of Bayesian estimation are briefly discussed in the section on parameter estimation.

**Table 1:** Major methods for variance component estimation in a mixed model framework.

Category	Method (abbreviation)	Property
ANOVA-based estimation	Henderson’s method 3	Unbiasedness Possibility of negative estimate (e.g., out of parameter space)
		Unknown distribution
		Lack of uniqueness
Distribution-free quadratic estimation	Minimum norm quadratic unbiased estimation (MINQUE)	No normality assumption
	Iterative MINQUE (I-MINQUE)	Possibility of negative estimate
		No normality assumption
		Asymptotic normality
		Possibility of negative estimate
	Minimum variance quadratic unbiased estimation (MIVQUE)	Equivalent to MINQUE with null priors (MINQUE0)
		Properties shared with MINQUE
Likelihood-based estimation	Maximum likelihood (ML)	Normality assumption
		Non-negative estimate by maximization within parameter space
		Asymptotic unbiasedness
		Asymptotic efficiency
		No closed form solution
	Restricted maximum likelihood (REML)	Explaining degrees of freedom involved in fixed effects
		Relatively free from normality assumption^1^
		Non-negative estimate
		Asymptotic unbiasedness
		Asymptotic efficiency
		No closed form solution
		Various numerical solutions are available
		The most popular method
Bayesian estimation	Gibbs sampling	Direct inference from posterior distribution^2^
	Metropolis and Hastings	Direct inference from posterior distribution^2^
		Data augmentation


*^1^REML was originally derived under normality assumption as ML, but REML estimates can be corresponding to I-MINQUE, which does not require the normality assumption (Brown, 1976).*

*^2^Point (e.g., posterior mean) and interval (e.g., 95% credible interval) estimates are directly obtained using the samples of posterior distribution generated by a Markov chain Monte Carlo.*

Forming a covariance structure of random effects is a critical step for a mixed model analysis. The structure could be generated as a matrix with elements of pairwise genetic relationships among individuals based on pedigree information. It was first called a numerator relationship matrix, and efficient algorithms for building and inverting the matrix enabled geneticists to handle large matrices (Quaas, 1976, 1988). This matrix should be modified according to genetic model, and only a portion of the genetic variance can be explained by the analytical model with the matrix. For example, while an animal model explains all of the genetic variance (Quaas and Pollak, 1980), a sire model explains only a quarter (Wang et al., 1993), and a sire-maternal grand sire model explains 3/8 (Lee and Pollak, 2002).

The mixed model has been applied to GWAS (Kang et al., 2010; Zhang et al., 2010; Yang et al., 2011). The only difference is that genetic covariance between individuals is assessed by genotype information, instead of pedigree information. Genotype information for a large number of nucleotide sequence variants is available owing to dramatic improvements in sequencing technologies. The mixed model can be employed to analyze gene expression, instead of phenotypes.

In summary, mixed models and the corresponding estimation methodology have been improved since their development to explain the genetics of complex phenotypes. They were, of course, adapted for applications to particular disciplines, such as evolution (Wilson et al., 2006), ecology (Melbourne and Hastings, 2008), and social sciences (Bartholomew et al., 2008). Irrespective of their application, they have a covariance structure of random effects as a common characteristic and this common structure is the reason why mixed models are used. The covariance structure for the genetics of complex phenotypes is represented as the numerator relationship matrix constructed by various ways. In genome-wide eQTL analyses by mixed models, the numerator relationship matrix can be constructed using genome-widely available nucleotide variant data, explaining polygenic effects. Since polygenic effects reflect different genetic backgrounds among individuals, the mixed model analysis is a powerful method for identifying accurate eQTLs. Of course, it may avoid spurious eQTLs produced by confounding effects of population stratification and kinship. Note that “genomic similarity matrix” is used as the variant-based genetic relationship matrix hereafter in this review.

## Genome-Wide eQTL Analysis Using Mixed Models

A mixed model for genome-wide eQTL analyses is presented in a generalized form with matrices as follows:

y =Xβ+g+ε

where **y** is the *n* × 1 vector of gene expression levels, *n* is the number of the gene expression levels, β is the *n_f_* × 1 vector of fixed effects (e.g., gender, age, and nucleotide variant effects), *n_f_* is the number of the fixed effects, and **X** is the *n* × *n_f_* design matrix for the fixed effects. The fixed effects include the minor allele effect of the candidate nucleotide variant, and the corresponding column of **X** includes elements of 0, 1, and 2 for the homozygous major allele, heterozygous genotype, and homozygous minor allele under the assumption of an additive model with a biallelic nucleotide variant. **g** is the *n* × 1 vector of random polygenic effects $\left(g∼N\left(0,G\sigma_{g}^{2}\right)\right)$ where **G** is the *n* × *n* genomic similarity matrix with elements of pairwise genomic similarity coefficients based on genotypes of nucleotide variants, and $\sigma_{g}^{2}$ is the polygenic variance component. The genomic similarity coefficient between individuals *j* and *k* can be calculated as follows:

$$g_{ik}=\frac{1}{n_{v}}\sum_{i=1}^{n_{v}}\frac{(\tau_{ij}-2f_{i})\left(\tau_{ik}-2f_{i}\right)}{2f_{i}\left(1-f_{i}\right)}$$

where *n_v_* is the number of nucleotide variants that contribute to the genomic similarity, τ*_ij_* and τ*_ik_* represent the number (0, 1, or 2) of minor alleles for the nucleotide variant *i*, and *f_i_* is the frequency of the minor allele. ε is the *n* × 1 vector of random environmental effects $\left(\varepsilon ∼N\left(0,I\sigma_{\varepsilon }^{2}\right)\right)$, where I is the *n* × *n* identity matrix, and $\sigma_{\varepsilon }^{2}$ is the environmental variance component. Variance in gene expression is thus defined as $\mathrm{var}(y)=V=G\sigma_{g}^{2}+I\sigma_{\varepsilon }^{2}$.

To avoid underestimating the association of gene expression with the candidate nucleotide variant by proximal contamination, genomic similarity coefficients can be estimated by excluding nucleotide variants that are in linkage disequilibrium with the candidate. One strategy is to exclude all variants located on the same chromosome as the candidate variant (Lippert et al., 2011). Theoretically, different genomic similarity coefficients are required for evaluating associations with every nucleotide variant, but this strategy reduces the burden by estimating the genomic similarity matrix for the same number of chromosomes. This efficiency should be stressed because the computing and memory costs for the genomic similarity matrix based on nucleotide variant information are expensive, unlike dealing with the sparse genetic relationship matrix based on pedigree information.

Considering only independent variants with more than a certain effect size is another efficient way to substantially reduce the cost. Only representative variants based on linkage disequilibrium can be used to explain polygenic effects (Lippert et al., 2011). The excessive exclusion of variants, however, may lead to insufficient correction for stratification (Yang J. et al., 2014). An example for selecting representative variants is to maximize polygenic variance by a stepwise selection of variants in linkage equilibrium with *r*^2^ < 0.8. The selection process should be conducted for every gene and thus requires an expensive computing cost. Thus, it might be convenient to select variants (*r*^2^ < 0.8) with an arbitrary significance threshold (*P* < 0.05).

The variance components for polygenic and environmental effects are usually estimated by employing REML prior to estimating fixed and random effects. For example, variance components can be estimated by maximizing the log restricted likelihood (Harville, 1977; Searle, 1979) as follows:

$$l_{r}\infty -\frac{1}{2}\left(\log|G\sigma_{g}^{2}|+\sigma_{\varepsilon }^{2n}+|C|+y^{\prime }Py\right)$$

Where $P=V^{-1}-V^{-1}X{\left(X^{\prime }V^{-1}X\right)}^{-1}X^{\prime }V^{-1}$ and $C=\left(\begin{array}{cc} X\prime X & X\prime \\ X & 1+\frac{\sigma_{\varepsilon }^{2}}{\sigma_{g}^{2}}G^{-1} \end{array}\right)$, which is the coefficient matrix of Henderson’s mixed model equation (MME; Henderson et al., 1959):

$$\left(\begin{array}{cc} X\prime X & X\prime \\ X & 1+\frac{\sigma_{\varepsilon }^{2}}{\sigma_{g}^{2}}G^{-1} \end{array}\right)[\begin{array}{c} \beta \\ g \end{array}]=[\begin{array}{c} X\prime y\\ y \end{array}].$$

Any closed form solutions for variance components are unavailable because the likelihood is non-linear, and various computing algorithms for obtaining REML estimates of variance components have been suggested as a non-trivial task. The log likelihood function presented above is efficient for obtaining REML estimates as one of the simplified forms, especially for the derivative-free algorithm incorporating the Choleski decomposition and simplex method (Boldman and Van Vleck, 1991). For detailed descriptions of a variety of variance component estimation methods (see Searle et al., 2009).

The fixed and random effects are then solved with the estimated variance components under the MME. The heavy computational burden on inverse matrices for solving MME can be avoided by Choleski decomposition or by iteration methods, such as the Jacobi and Gauss–Seidel algorithms (Lee, 2016b).

The identification of eQTL is performed by a *t*-test with 1 degree of freedom for candidate variant effects. Since a large number of tests for associations with genome-wide nucleotide variants are usually conducted, adjustments for multiple testing are employed to avoid spurious eQTLs. The most common method is the Bonferroni correction under the assumption of independence among individual tests. Researchers often use a less conservative method (e.g., false discovery rate), especially for a huge number of tests. Regardless of the number of tests, significance threshold value of *P* = 5 × 10^-8^ is acceptable for GWAS (Dudbridge and Gusnanto, 2008; Jannot et al., 2015). If only *cis*-regulatory eQTLs are considered, a smaller significance threshold value can be used. For example, significance threshold values used for the *cis*-eQTLs within 1 Mb from the transcription start site were *P* = 2.82 × 10^-5^ (Koopmann et al., 2014) and *P* = 9.22 × 10^-5^ (Gong et al., 2017). The selection of eQTLs for polygenic random effects might be distinguished from the eQTL identification addressed above. While the identification of eQTLs focuses on avoiding spurious eQTLs, the selection of eQTLs focuses on the appropriate reflection of polygenic effects. Thus, eQTLs might be selected without any correction for multiple testing.

## Advantages of Mixed Models for eQTL Analyses

The mixed model framework not only enables the identification of eQTLs by determining the statistical significance of associations with gene expression, but also shows polygenetic variance explained by nucleotide variants. Thus, genome-wide eQTL analyses using the mixed model may substantially reduce “missing heritability,” which is usually attributed to inherent difference between GWAS and pedigree-based genetic analyses. Furthermore, the element of vector g indicates the relative genetic ability of each individual for gene expression.

The use of a genomic similarity matrix would help to control for population stratification, to explain polygenic effects, and thus to reduce false positive and negative genetic associations. The mixed model analysis for data simulated with a variety of designs performed better than the fixed model analysis incorporating genomic control or principal component analysis in respect to empirical type 1 error rate and statistical power (Widmer et al., 2014; Shin and Lee, 2015a). The improvement by the mixed models increased more with a highly admixed population, a large narrow-sense heritability, a small number of causal variant, or a large number of related individuals (Widmer et al., 2014; Shin and Lee, 2015a,b).

The assumption of an infinitesimal model is not required for the identical-by-state (IBS) genetic relationship (i.e., genomic similarity matrix) based on genotype information, unlike for the identical-by-descent genetic relationship based on pedigree information. That is, the IBS genetic relationship matrix can be flexibly constructed using genotype information for a customized set of selected nucleotide variants. This is useful for eQTL mapping where the cell-specific genomic similarity matrix should be constructed with different loci. Gene expression is regulated by the cell environment, and the cell environment is produced by gene expression regulation. Thus, trans-regulators as well as *cis*-regulators should be stressed to construct the genomic similarity matrix, and different genomic similarity matrices among cells are largely attributed to cell-specific trans-regulators. Cell-specific genomic similarity increases the accuracy of eQTL identification and heritability estimates explained by eQTLs. Gene-specific similarity is also required because the loci used to estimate the genomic similarity matrix vary widely in kind and size depending on gene functions. The selection of loci is determined by statistical significance for associations with gene expression using a specific significance threshold. A subjective significance threshold is employed, or the value is determined by maximizing polygenic variance estimated with the selected loci. In conclusion, a cell- and gene-specific genomic similarity matrix should be constructed for eQTL analyses, without the unjustifiable assumption of an infinitesimal model.

Using mixed models, it is feasible to extend the additive genetic analysis presented in this review to non-additive genetic analyses. For example, an analytical model may include random dominance polygenic effects with corresponding variances, e.g., a dominance genomic similarity matrix multiplied by dominance genetic variance (Da et al., 2014). Similarly, additive-by-additive, additive-by-dominance, dominance-by-dominance, and/or higher order epistatic terms can be added, each with their own variance, e.g., $G^{2}\sigma_{g\times g}^{2}$ (Martini et al., 2016). However, careful modeling is required for epistatic analyses because unreliable results are more likely as the degree of interaction increases. Filtering according to biological relevance-, gene module-, and marginal effect-based strategies may avoid exhaustive searches for epistasis (Huang et al., 2013). Filtering also helps to overcome another challenge arising from a large number of weak signals.

Mixed models enable the partitioning of the polygenic variance by its subsets as sell as the estimation of polygenic variance with any customized set of nucleotide variants. For example, polygenic variance may be partitioned by nucleotide variants proximal and distal to the gene of interest in order to infer that they are *cis*- and *trans*- eQTLs, respectively. These *cis*- and *trans*- eQTLs might be interpreted further as potential global and cell-specific regulators (Thalayasingam et al., 2018).

## Issues with Analytical Models

Simulation studies have shown that mixed models perform well, regardless of the use of IBD or IBS genetic relationships, showing empirical unbiasedness (Lee and Pollak, 1997a; Ryoo and Lee, 2014). The fitness of the analytical model employed for analyzing real data must be confirmed prior to the analysis. If the normality assumption is violated for gene expression, data transformations should first be considered, such as normalization and log-transformation. Alternatively, more flexible distributions might be assumed for analytical models, such as generalized linear mixed models (Breslow and Clayton, 1993) and hierarchical generalized linear models (Lee and Nelder, 1996).

The analytical model presented in this review assumes a consistent effect for every eQTL in assessing the genetic covariance structure among individuals. This unrealistic assumption might produce bias in the genetic variance component (Ryoo and Lee, 2014). Thus, heterogeneous effects can be incorporated into the model. For example, the genomic similarity may reflect a penalty based on functions for each eQTL effect size (Yi and Xu, 2008), and a Bayesian approach with priors on the number of major eQTLs is also plausible (Lee et al., 2008).

When analyzing gene expression, it is possible to use gene expression at a previous stage as a covariate in order to determine the regulatory stage. For example, an eQTL for protein level might result from the regulation of transcription or translation. This is tested by employing an analytical model with the protein level as a dependent variable and RNA level as a covariate. The test can be used to confirm a candidate gene for a phenotype by employing an analytical model with phenotype as a dependent variable and its expression level as a covariate, especially when the candidate gene (i.e., the nearest gene to the association signal) obtained by GWAS differs from the gene identified by the eQTL analysis. Paired *t*-tests with expression data obtained at two stages for every individual can avoid inflating the sampling variance for statistics estimated from the separate analysis of independent two-stage expression data for unrelated individuals. Such joint modeling also provides the proportion of phenotypic variance explained by all eQTLs identified for the expression of a specific candidate gene (Huang et al., 2014).

Sex effects might be simply treated as a covariate in the analytical model. However, heterogeneous effects of sex are also important as an interaction between eQTLs and sex, especially for genes with hormone-dependent functions. Heterogeneous eQTLs by sex can be explained by separate analyses with partitioned data or by sex-stratified bivariate mixed models (Lee, 2016a). A straightforward sex-stratified model is the two-phenotype model in which male expression is treated as one phenotype and female expression as the other (Lee et al., 1997, 2012). The heterogeneous eQTLs by sex clarify the heterogeneous genetic architecture with respect to sex for various complex phenotypes.

Joint modeling in the mixed model framework can be extended to analyze various kinds of expression simultaneously. For example, expression data for multiple tissues can be treated as different phenotypes, and these analyses provide genetic covariance components and genetic correlations between tissues. A mixed model meta-analysis can be applied to the identification of eQTLs with heterogeneous effects across multiple tissues (Sul et al., 2013).

Spurious eQTLs easily result from microarray expression data because confounding effects are induced by various measurement errors (Churchill, 2002; Akey et al., 2007). Mixed model analyses, such as the intersample correlation emended method (Kang et al., 2008), probabilistic analysis of genomic data (Fusi et al., 2012), and confounding factor estimation through independent component analysis (Ju et al., 2017), have been suggested to correct for the confounding effects. These analytical models incorporate random effects with an intersample covariance structure that might explain unknown confounding factors produced by measurement errors.

## Issues Related to Parameter Estimation

The mixed model is usually applied with the statistical property of best linear unbiased estimator (BLUE, a solution of fixed effects) and best linear unbiased predictor (BLUP, a solution of random effects) derived from Henderson’s mixed model equations. The BLUE and BLUP are, however, only valid under the assumption of known variance components. In reality, variance components are unknown for specific data, and should be estimated with the data used for BLUE and BLUP. This contradictory assumption has not limited its practical application. This might be because employing REML works well for the practical estimation of variance components. In order to actively address with the problem, BLUE can be estimated simultaneously with the variance and covariance components by a Bayesian approach implemented with MCMC methods, such as Gibbs sampling and the Metropolis and Hastings algorithm (Gilks et al., 1995). Another advantage of Bayesian methods is that they reflect uncertainty in unknown parameters, such as variance components for the analytical model, by treating the parameters as random variables. As a result, the Bayesian approaches provide a probability distribution called the posterior for each parameter. This enables us to make straightforward inferences for the parameters. For example, specific credible intervals for every parameter considered in an analytical model can be directly obtained using the samples of posterior distribution generated by the MCMC. The posterior avoids doubts about undesirable local ML estimates produced from a frequentist approach. In practice, a search for the maximum of a likelihood function is mathematically and computationally challenging.

## Software

Mixed model methods for GWAS have been implemented with a variety of software. Most of them provide REML and Bayesian estimates of parameters, and some useful software for genome-wide eQTL analysis are presented in **Table 2**. In particular, GEMMA and TASSEL employed the Newton–Raphson algorithm using observed Fisher information matrix (i.e., Hessian matrix) as the second derivative of likelihood for REML (Zhang et al., 2010; Zhou and Stephens, 2014), and GCTA and MTG2 employed the average information algorithm using both of the Hessian matrix and Fisher information matrix (Yang et al., 2011; Lee and van der Werf, 2016). The algorithms were both used in MMAP (O’Connell, 2014).

**Table 2:** Useful software for genome-wide eQTL analysis using mixed models.

Program	Method and algorithm	Website (http)	MA^1^	Source code	Reference
GCTA	Average information restricted maximum likelihood (AIREML)	cnsgenomics.com/software/gcta	Δ	C++	Yang et al., 2011
GEMMA	Newton–Raphson restricted maximum likelihood (NRREML) Bayesian using Metropolis and Hastings	www.xzlab.org/software.html	O	C++	Zhou and Stephens, 2014 Zhou et al., 2013
TASSEL	NRREML	www.maizegenetics.net/tassel	X	Java	Zhang et al., 2010
MTG2	AIREML	sites.google.com/site/honglee0707/mtg2	O	FORTRAN	Lee and van der Werf, 2016
GENSEL	Bayesian using Gibbs sampling	archive.is/bigs.ansci.iastate.edu	X	C++	Kizilkaya et al., 2010
MMAP	AIREML, NRREML, Expectation-maximization restricted maximum likelihood, Fisher information restricted maximum likelihood	mmap.github.io	X	Undisclosed	O’Connell, 2014
FaST-LMM	Maximum likelihood^2^, Restricted maximum likelihood^2^	www.microsoft.com/en-us/research/project/fastlmm	X	Python	Lippert et al., 2011


*^1^Multivariate analysis; Δ indicates that bivariate analysis is available.*

*^2^Spectral decomposition-based algorithm is employed.*

## Closing Remarks

**FIGURE 1:**
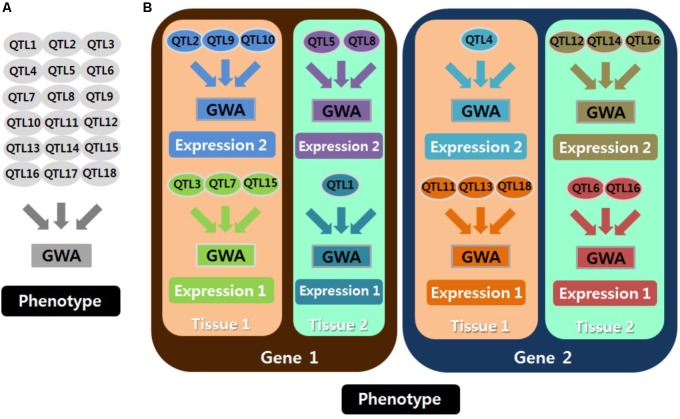
Schematic concepts for analyzing quantitative trait locus (QTL) associated with phenotypes **(A)** and with gene expression **(B)**. Each genome-wide association analysis (GWAS) is presented in a different color. Many GWAS should be conducted for QTL associated with gene expression. Thus, employing a mixed model is important because every GWA requires a different genomic similarity matrix, and the cellular environment for gene expression is largely the result of genetic effects. Expression 1 and expression 2 are distinguished by different kinds or stages of expression (e.g., RNA expression and protein expression).

Mixed models are important for GWAS to explain polygenic effects and thus to avoid population stratification. In particular, polygenic effects for gene expression might be more sensitive than those for phenotypes. This is because the cellular environment for gene expression results largely from genetic effects, and noise produced by a long process from genotype to phenotype is decreased in gene expression analyses. Furthermore, accurate analyses are essential to identify specific regulatory stages and functions of eQTLs for gene expression, and eQTLs can be specified with the corresponding technique, i.e., chromatin modification eQTL (Degner et al., 2012; Grubert et al., 2015), including DNase I sensitivity QTL (dsQTL), methylation QTL (meQTL), and histone QTL (hQTL); transcriptional eQTL (Lappalainen et al., 2013; Li et al., 2016), including narrow-sense eQTL, splicing QTL (sQTL), transcript ratio QTL (trQTL), miRNA QTL (mirQTL), allele specific expression QTL (aseQTL), RNA synthesis rate QTL (rsQTL), and RNA decay QTL (rdQTL); chromatin interaction eQTL (Tang et al., 2015), including chromatin interaction QTL (cQTL) and promoter enhancer interaction QTL (peQTL); and translational eQTL (Battle et al., 2015), including ribosome occupancy QTL (rQTL) and protein abundance QTL (pQTL). The accuracy of eQTL identification and parameter estimation depends on the customized genomic similarity matrix for each genome-wide analysis by expressed molecules and tissues as well as by genes (**Figure 1**). Of course, the specified analyses can be extended to the identification of any other potential heterogeneity in eQTLs. An example is age-dependent eQTLs, which may explain the heterogeneous heritability of complex phenotypes by age (Lee and Lee, 2015). Thus, employing a mixed model should be emphasized to reduce spurious eQTLs in genome-wide eQTL analyses.

The use of the mixed model for genome-wide eQTL analyses provides more reliable results than conventional fixed model analyses. Of course, accuracy will be further improved by decreasing errors produced from current RNA-seq techniques and costs (e.g., sequencing death). Understanding the genetic architecture of complex phenotypes will be accelerated by genome-wide eQTL analyses using mixed models with a profile of transcriptome-wide gene expression for the activity of a single cell and further with multiple profiles across cells with different functions.

## Author Contributions

Theauthor confirms being the sole contributor of this work and approved it for publication.

## Conflict of Interest Statement

The author declares that the research was conducted in the absence of any commercial or financial relationships that could be construed as a potential conflict of interest.
